# Association of individual-based morphological brain network alterations with cognitive impairment in type 2 diabetes mellitus

**DOI:** 10.3389/fneur.2024.1519397

**Published:** 2025-01-09

**Authors:** Die Shen, Xuan Huang, Ziyu Diao, Jiahe Wang, Kun Wang, Weiye Lu, Shijun Qiu

**Affiliations:** ^1^The First Clinical Medical College, Guangzhou University of Chinese Medicine, Guangzhou, China; ^2^Department of Radiology, The First Affiliated Hospital of Guangzhou University of Chinese Medicine, Guangzhou, China; ^3^State Key Laboratory of Traditional Chinese Medicine Syndrome, Guangzhou, China

**Keywords:** type 2 diabetes mellitus, cognitive impairment, graph theory, morphological brain network, structural magnetic resonance imaging

## Abstract

**Objective:**

To investigate the altered characteristics of cortical morphology and individual-based morphological brain networks in type 2 diabetes mellitus (T2DM), as well as the neural network mechanisms underlying cognitive impairment in T2DM.

**Methods:**

A total of 150 T2DM patients and 130 healthy controls (HCs) were recruited in this study. The study used voxel- and surface-based morphometric analyses to investigate morphological alterations (including gray matter volume, cortical thickness, cortical surface area, and localized gyrus index) in the brains of T2DM patients. Then two methods, Jensen-Shannon divergence-based similarities (JSDs) and Kullback–Leibler divergence-based similarities (KLDs), were used to construct individual morphometric brain networks based on gray matter volume, to discover altered features of the topological network and extract abnormal key brain regions. Subsequently, partial correlation analyses were performed to explore the relationship between clinical biochemical indices, neuropsychological test scores, and altered cortical morphology and network indices.

**Results:**

Brain regions with reduced gray matter volume and cortical thickness in T2DM patients were mainly concentrated in the frontal lobe, temporal lobe, parietal lobe, anterior cingulate gyrus, insula, lingual gyrus, and cerebellar hemispheres. The global attributes of the Individual-based morphological brain network were significantly reduced (Cp, Eloc, *σ*), with an increase in the nodal efficiency of the hippocampus and the nodal local efficiency of the anterior cingulate gyrus, and the nodal local efficiency of the parahippocampal gyrus and transverse temporal gyrus were reduced. There was a correlation between these node attributes and cognitive scale scores.

**Conclusion:**

This study demonstrated that patients with T2DM exhibit generalized cortical atrophy and damage to individual morphologic brain networks. It also identified overlapping and cognitively relevant key brain regions, primarily within the limbic/paralimbic network (especially the hippocampus and cingulate gyrus), which may serve as imaging markers for identifying cognitive deficits in T2DM. These findings offer new insights into the neural network mechanisms underlying T2DM-associated brain damage and cognitive impairment.

## 1 Introduction

Diabetes mellitus, a serious chronic disease, is characterized by elevated blood glucose concentrations associated with islet *β*-cell dysfunction (1). The global incidence of diabetes is escalating rapidly, with projections indicating a dramatic rise to 1.31 billion affected individuals by 2050, and prevalence exceeding 10% in some regions, predominantly driven by type 2 diabetes mellitus (T2DM), which constitutes over 95% of diagnosed cases (2, 3). This escalating health crisis poses significant challenges to human well-being and economic stability.

T2DM can lead to multi-system damage (4), including brain disorders such as stroke, depression, cognitive dysfunction, and dementia, which are considered important complications of T2DM (5). Diabetes mellitus is a risk factor for the development of dementia (6–8), with some studies suggesting that the risk of cognitive dysfunction in patients with type 2 diabetes mellitus is 1.5 to 2.0 times higher than that of non-diabetic patients (9, 10). Early identification of cognitive deficits in T2DM and timely intervention can reduce the number of people developing dementia (11). Consequently, exploring the neurobiological underpinnings of cognitive impairments in T2DM is essential for facilitating timely clinical identification and targeted interventions.

In recent years, many neuroimaging studies have revealed alterations in local gray matter microstructure and neural activity in T2DM (12, 13). Common cortical morphology research methods mainly include voxel-based and surface-based morphology (VBM and SBM). Some studies have suggested that VBM and SBM should be used simultaneously as complementary methods for detecting cortical morphology changes to obtain more information (14). It has also been found that T2DM is associated with large-scale brain network abnormalities (15). The brain is a highly efficient and interconnected network system, and more information can be discovered by constructing neural networks. Studies utilizing functional magnetic resonance imaging (fMRI) (16–18) and diffusion MRI (DTI) have revealed changes in the topological properties of brain networks in T2DM (15, 19).

Beyond the two types of networks mentioned, morphological brain networks derived from structural MRI data offer a complementary approach to probing human brain network characteristics. However, the traditional structural covariance network is based on the structural information (gray matter volume or cortical thickness, etc.) of a group of people to construct a network, which can only reflect the group-level brain morphological characteristics and neglects the inter-individual variability. This restricts its application to the investigation of brain structure in terms of individual variability, especially in identifying structural brain abnormalities in a single patient. The recently proposed individual-level morphological similarity network approach can effectively resolve the above limitations (20). This method employs Jensen–Shannon divergence-based similarity (JSDs) to quantify the morphological similarity between distinct brain regions (21), offering a personalized assessment. This approach has been widely applied to many disorders, including depression (22), rolandic epilepsy (23), attention deficit hyperactivity disorder (24), and spinal cerebellar ataxia (25). As far as we all know, the application of structural MRI-based individual-level morphological network analysis for investigating brain network alterations in T2DM remains unreported.

In the present study, we aimed to characterize the altered brain cortical morphology in T2DM patients based on VBM and SBM analysis, to explore the topological changes in Individual-based morphological brain network in T2DM, to identify the key brain regions from them, and to investigate further these changes about clinical indicators and cognitive correlations.

## 2 Materials and methods

### 2.1 Participants

In this investigation, patients diagnosed with T2DM were enrolled in the Department of Endocrinology at the First Affiliated Hospital of Guangzhou University of Traditional Chinese Medicine between October 2021 and December 2023. The diagnosis of T2DM adhered to the criteria established by the American Diabetes Association (26). Concurrently, we selected healthy controls (HCs) from the community, ensuring they were age-, gender-, and education-level-matched to the T2DM cohort. The age range for all participants was confined to 35 to 70 years, and all were right-handed.

Exclusion criteria for all participants included (1) any other neurological or psychiatric disorders such as epilepsy, depression, and so on; (2) the presence of endocrine disorders such as hyperthyroidism, hypothyroidism, Cushing’s syndrome, etc.; (3) significant parenchymal brain lesions including cerebral hemorrhage, cerebral ischemic stroke, or brain tumors; (4) left-handedness; and (5) contraindications to MRI scanning.

Conclusively, the study enrolled 150 patients with T2DM and 130 healthy participants. Ethical approval for the research was secured from the Medical Research Ethics Committee of our hospital, with all participants furnishing written informed consent before their engagement in the study protocol.

### 2.2 Clinical and neuropsychological measurements

We systematically recorded height, weight, body mass index (BMI), and arterial blood pressure in all subjects. In addition, we recorded the duration of the disease in patients with T2DM. Comprehensive laboratory assessments including glycosylated hemoglobin (HbA1c), fasting blood glucose (FBG), fasting insulin (FINS), total cholesterol (TC), triglycerides (TG), and low-density lipoprotein (LDL) were performed in patients with T2DM and healthy participants. The Homeostasis Model Assessment of Insulin Resistance (HOMA-IR) was calculated as HOMA-IR = [FINS (μIU/mL)] × [FBG (mmol/L)] / 22.5.

Furthermore, all participants underwent a battery of cognitive evaluations, comprising the Montreal Cognitive Assessment (MoCA) (27), Mini-Mental State Examination (MMSE) (28), Auditory Verbal Learning Test (AVLT) (29), Grooved Pegboard Test (GPT) (30), Digit Span Test (DST) (31), Clock Drawing Test (CDT) (32), Digit Symbol Substitution Test (DSST) (33), and Trailblazer Test-A (TMT) (34).

### 2.3 MRI data acquisition

MRI data acquisition was performed utilizing a Siemens MAGNETOM Prisma 3.0 Tesla MRI scanner equipped with a 64-channel head coil. T2-weighted and T2-FLAIR imaging sequences were employed for the detection and exclusion of organic brain lesions. The 3D T1-weighted imaging (T1WI) sequence parameters for neuroimaging analysis were as follows: inversion time of 1,100 ms, repetition time of 2,530 ms, echo time of 2.98 ms, flip angle of 7°, field of view of 256 × 256 mm^2^, slice thickness of 1.0 mm, 192 slices acquired, and voxel dimensions of 1.0 × 1.0 × 1.0 mm^3^. Participants were directed to keep their eyes closed and remain conscious throughout the scanning procedure.

### 2.4 Data processing

Voxel-based morphometry analysis (VBM) was conducted using the Computational Anatomy Toolbox 12 (CAT12)^1^ within the Statistical Parametric Mapping 12 (SPM 12)^2^ framework. The analytical pipeline commenced with quality assurance of the image datasets, followed by spatial normalization to the Montreal Neurological Institute (MNI) space. Subsequently, the images were segmented into gray matter (GM), white matter (WM), and cerebrospinal fluid compartments. Jacobian modulation was applied to generate GM volume maps, which were subsequently smoothed with an isotropic Gaussian kernel (half-width at half-maximum = 6 mm) for statistical analysis. Finally, a report was generated containing a weighted average rating (IQR) of the image quality, with an IQR score greater than 80, considering the image to be of good quality.

Surface-based morphological analysis (SBM) was computed on T1-weighted MRI data using FreeSurfer version 7.3.2.^3^ The main steps included motion correction, cranial stripping, field anisotropy correction, alignment in Talairach coordinate space, etc. The cortex was segmented into 68 brain regions using the Desikan-Killiany atlas (DK68), and the cortical thickness, surface area, and local gyrification index (LGI) were extracted and computed for each brain region of each subject and then smoothed using a Gaussian kernel with a full width at half-maximum (FWHM) of 15 mm.

### 2.5 Construction of an individual morphological brain network

Individual morphological similarity networks were constructed based on Matlab 2022b script files. Firstly, the brain was first segmented into 90 brain regions using the automated anatomical atlas (AAL90) (35) and each brain region was defined as a node in the network, the GMV values of each brain region were extracted, and then the similarity of the GMV distributions among the brain regions was calculated as the edges of the network (36). The calculation was performed by first calculating the probability density function of the 90 GMV values for each subject using kernel density estimation, then further calculating the probability density function as a probability distribution function (PDF), and then quantifying the connectivity of the PDF morphology of the two regions using Jensen-Shannon divergence-based similarity (JSD) (21) to construct a 90 × 90 similarity matrix. In addition, Kullback–Leibler divergence-based similarities (KLDs) (37) is another method for calculating individual similarity brain networks, and like JSD, both have been shown to be robust and sensitive methods (38), and we used the KLD method to test the reliability and reproducibility of the experimental results.

### 2.6 Network metrics

Network properties were calculated using the GRETNA toolbox^4^ in MATLAB 2022b.^5^ The global topological metrics encompassed the clustering coefficient (Cp), characteristic path length (Lp), normalized clustering coefficient (*γ*/Gamma), normalized characteristic path length (*λ*/Lambda), small-world parameters (*σ*/Sigma), global efficiency (Eg), local efficiency (Eloc). Global attributes reflect the efficiency and degree of integration of the network as a whole. Nodal topological metrics include nodal efficiency (Ne), degree centrality (DC), nodal local efficiency (NLe), and betweenness centrality (BC). Node attributes reveal the role and importance of individual nodes in the network. The sparsity threshold (S) is defined as the ratio of the number of available edges to the maximum possible number of edges. Adopting a wide sparsity range reduces spurious connectivity between nodes and ensures that the small-world index is >1.0. In our study, we set the sparsity scope between 0.05 and 0.4 with an interval of 0.01 (39), with a total of 36 thresholds, and computed the area under the curve (AUC) of each metric within the sparsity range for subsequent statistical analyses.

### 2.7 Statistical analysis

The statistical analyses were performed using the Statistical Package for the Social Sciences (IBM SPSS 27.0) to analyze demographics, clinical indicators, and cognitive scale information. Continuous variables were tested for normality using the Shapiro–Wilk test; parametric two-sample t-tests were used for normally distributed data, otherwise non-parametric tests were used. Categorical variables were assessed using the chi-square test, with statistical significance set at two-tailed *p* < 0.05.

Two-sample t-tests for smoothed GMV were performed using SPM12 with age, sex, education, and total intracranial volume (TIV) as covariates. The results were corrected by cluster-wise family-wise error (FWE) correction with *p* < 0.001 and cluster number > 521.

Statistical inferential analyses were performed using the FreeSurfer (7.3.2) tool and general linear modeling (GLM) was used for between-group comparisons. We applied a cluster-wise correction for multiple comparisons and Monte-Carlo simulation corrected cluster thresholds of *p* < 0.05 for bidirectional effects. Covariates included age, sex, and education in all models; TIV was excluded because it was definitively correlated with head-size scaling, but not with thickness (40).

A two-sample t-test based on Gretna software was used to compare the area under the curve (AUC) of global and nodal network metrics between groups, and nodal metrics were corrected using the false discovery rate (FDR) with a significance value of 0.05.

Finally, the partial correlation coefficient was performed to calculate the correlation between significant global and nodal network indicators, morphological changes and cognitive scores, and clinical indicators in the T2DM group, with age, gender, education, and TIV as covariates, with statistical significance indicated by *p* < 0.05.

## 3 Results

### 3.1 Demographic information and clinical characteristics

Table 1 demonstrates the demographic details, cognitive scale scores, and clinical profiles of 150 individuals with T2DM and 130 healthy controls. No significant disparities in age, gender, and educational attainment were observed between the groups. T2DM patients exhibited diminished cognitive performance across the MoCA, MMSE, AVLT-recall, and GPT, with these differences attaining statistical significance (*p* < 0.05). Moreover, T2DM patients presented with elevated levels of HbA1c, FBG, TC, and FINS, all of which were significantly different compared to the control group (*p* < 0.05).

**Table 1:** Demographic and clinical characteristics of the participants.

Variable	T2DM (*n* = 150)	HC (*n* = 130)	Statistics (T/Z/*χ*2)	*p*-value
Age (years)	50.54 ± 8.70	51.02 ± 9.13	0.446	0.656
Gender (male/female)	88/62	70/60	0.658	0.417
Education (years)	12 (9, 14)	10 (9, 14)	−1.27	0.206
Duration (years)	5 (2, 10)	–	–	–
SBP (mmHg)	125.20 ± 14.89	127.59 ± 15.77	1.305	0.193
DBP (mmHg)	83.35 ± 10.06	83 (76.00, 89.25)	−0.061	0.952
BMI (kg/m^2^)	24.10 ± 3.34	23.44 (21.90, 25.73)	−0.569	0.569
HbA1c (%)	9.30 (8.50, 10.7)	5.70 (5.50, 5.90)	−14.090	**<0.001**
FBG (mmol/L)	8.52 (6.88, 10.50)	5.14 (4.83, 5.44)	−12.594	**<0.001**
FINS (μIU/mL)	5.99 (3.27, 10.94)	8.75 (6.32, 13.26)	−4.532	**<0.001**
HOMA-IR	2.21 (1.09, 5.18)	1.83 (1.19, 3.01)	−2.002	**0.045**
TG (mmol/L)	1.78 (1.23, 2.65)	1.27 (0.92, 1.74)	−4.816	**<0.001**
TC (mmol/L)	4.91 ± 1.09	5.01 (4.49, 5.73)	−2.288	**0.022**
LDL (mmol/L)	3.11 ± 0.96	3.28 (2.85, 3.88)	−2.279	**0.023**
MoCA	26 (23, 28)	27 (27, 28)	−0.431	**<0.001**
MMSE	28 (27, 29)	29 (27.75, 30.00)	−0.328	**0.001**
AVLT (immediate)	21.35 ± 5.13	21.84 ± 4.95	−0.857	0.391
AVLT (5 min)	8 (6, 10)	8 (7, 10)	−0.145	0.885
AVLT-delay	8 (6, 10)	8 (6, 10)	−0.617	0.537
AVLT-recall	11 (10, 12)	12 (11, 12)	−2.605	**0.009**
GPT (R)	73.04 (65, 86)	69 (60.00, 77.25)	−3.500	**<0.001**
GPT (L)	81.50 (71.00, 93.25)	73 (65.00, 83.25)	−4.430	**<0.001**
TMT-A	45 (35, 60)	44 (33.00, 60.25)	−0.660	0.509
CDT	4 (3, 4)	4 (3, 4)	−1.325	0.185
DSST	41.5 (31, 50)	40 (31.75, 51.00)	−0.498	0.618
DST (forward)	8 (6, 8)	7 (6, 8)	−0.081	0.936
DST (backward)	4 (3, 5)	4 (3, 5)	−1.296	0.195

Data are expressed as median (Q1, Q3) and mean ± SD, gender: male/female. Comparisons between groups were made using two-sample t-tests for normally distributed data, non-parametric tests for non-normally distributed data, and chi-square tests for gender, with bold values representing statistically significant (*p* < 0.05).BMI, body mass index; SBP, systolic blood pressure; DBP, diastolic blood pressure; HbA1c, hemoglobin A1c; FBG, fasting blood glucose; FINS, fasting insulin; HOMA-IR, static model assessment of insulin resistance; TG, triglycerides; TC, total cholesterol; LDL, low-density lipoprotein; MoCA, Montreal cognitive assessment; MMSE, mini-mental state examination; AVLT, Auditory Verbal Learning Test; GPT, grooved pegboard test; TMT-A: trail making test-A; DSST: digit symbol substitution test; CDT, clock drawing test; DST, digit span test.

### 3.2 VBM and SBM analysis results

**Figure 1:**
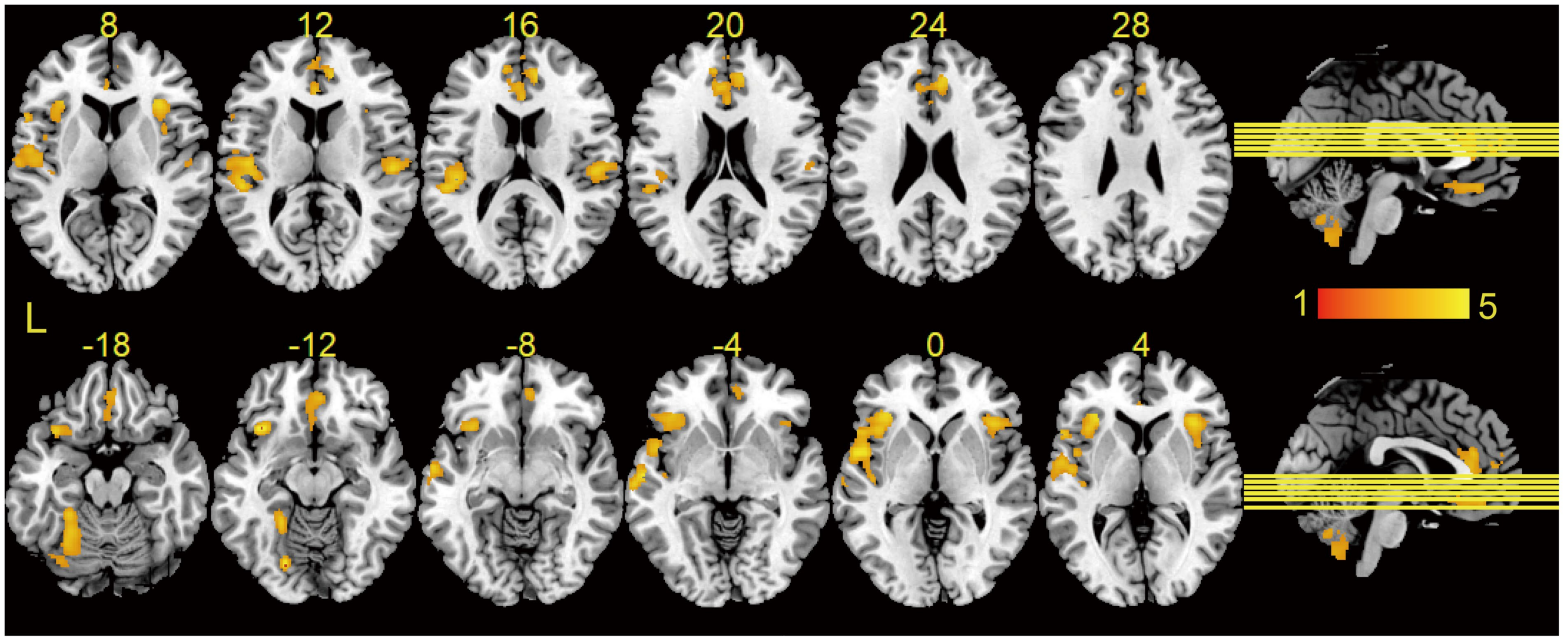
Results of VBM analysis between groups. Brain regions showed significantly decreased GMV in T2DM as compared to HCs using two-sample t-tests, and the results were corrected by cluster-wise family-wise error (FWE) correction with *p* < 0.001 and cluster number > 521. Brain regions without increased gray matter volume in T2DM. L, left.

Gray matter volume was significantly lower in T2DM patients than in HCs, and these regions mainly included: the left lingual gyrus, the left orbitofrontal inferior gyrus, the right anterior cingulate gyrus, the right insula, the right orbitofrontal middle gyrus, the right rolandic operculum, and the cerebellar hemispheres (Table 2; Figure 1), and most of these brain regions were concentrated in the central control network. However, no regions of increased gray matter volume were found in patients with T2DM.

**Table 2:** Results of gray matter volume reduction in the T2DM group.

Brain region	Cluster size	MNI coordinates, *x*, *y*, *z*	Peak intensity	*p*-value
Lingual_L	3,780	−20, −76, −12	5.364	0.000
Frontal_Inf_Orb_L	3,763	−36, 20, −14	5.437	0.000
Cerebelum_6_R	2,638	38, −57, −28	4.557	0.000
Cingulum_Ant_R	1,110	10, 45, 15	4.768	0.001
Cerebelum_9_L	842	−6, −62, −46	4.342	0.003
Insula_R	766	34, 24, 6	4.839	0.005
Frontal_Med_Orb_R	653	3, 39, −14	4.214	0.011
Rolandic_Oper_R	521	51, −21, 15	5.158	0.028

Using the anatomical automatic labeling (AAL) template; MNI, Montreal Neurological Institute; Lingual_L, left lingual gyrus; Frontal_Inf_Orb_L, left orbitofrontal inferior gyrus; Cingulum_Ant_R, right anterior cingulate gyrus; Insula_R, right insula; Frontal_Med_Orb_R, right orbitofrontal middle gyrus; Rolandic_Oper_R, right rolandic operculum; Cerebelum_6_R, right cerebellar hemisphere 6; Cerebelum_9_L, left cerebellar hemisphere 9. L, left; R, right.

**Figure 2:**
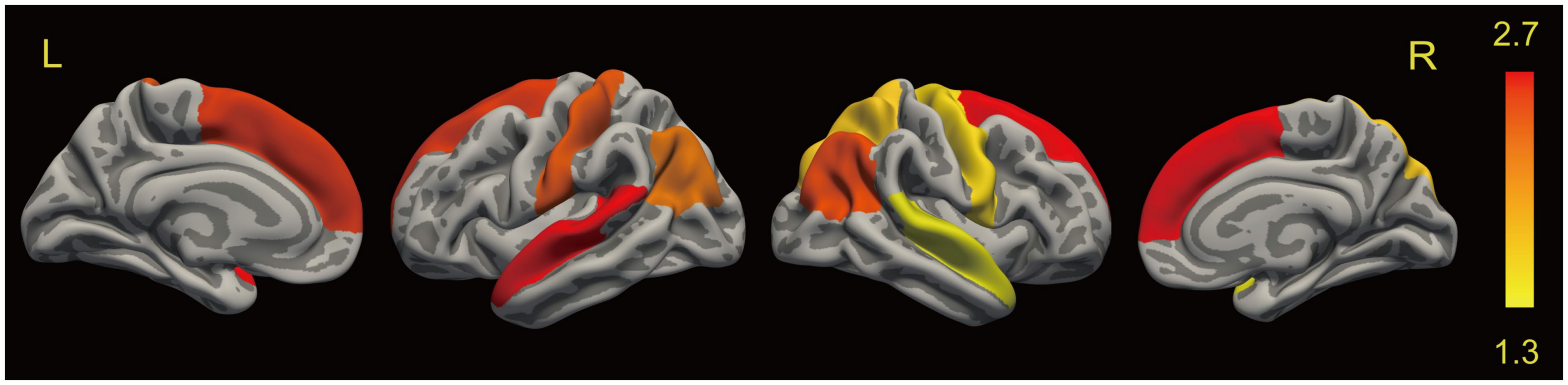
Results of SBM analysis between groups. Brain regions showed significantly decreased cortical thickness in T2DM as compared to HCs using the general linear model (GLM), and we applied a cluster-wise correction for multiple comparisons and Monte-Carlo simulation corrected cluster thresholds of *p* < 0.05 for bidirectional effects. No differences were found in surface area and LGI between groups. L, left; R, right.

Compared with HCs, patients with T2DM showed reduced cortical thickness in the bilateral superior frontal gyrus, precentral gyrus, inferior parietal lobule, superior temporal gyrus, and right parietal lobule (Table 3; Figure 2). However, there were no statistically significant differences in cortical surface area and LGI between the two groups. The statistical analyses above controlled for age, gender, and education to minimize their impact on the study results.

**Table 3:** Results of cortical thickness reduction in the T2DM group.

Sphere	Brain region	Size(mm^2^)	RAS coordinates, *x*, *y*, *z*	*p*-value
rh	Superior frontal	1123.63	13.6, 36.2, 48.1	0.002
rh	Inferior parietal	475.5	37.6, −48.8, 37.5	0.010
rh	Precentral	294.07	58.7, −4, 15.1	0.047
rh	Superior parietal	293.21	27.1, −43.5, 57	0.047
rh	Superior temporal	291.14	43.7, −34.5, 11.9	0.049
lh	Superior temporal	1071.28	−52.6, −33.1, 7.1	0.002
lh	Superior frontal	760.71	−7.8, 37, 50.4	0.004
lh	Precentral	667.97	−54.3, 4.5, 20.1	0.006
lh	Inferior parietal	549.12	−42.4, −61.1, 42.9	0.008

rh, right hemisphere; lh, left hemisphere.

### 3.3 Alterations in global profiles of morphological brain network

**Figure 3:**
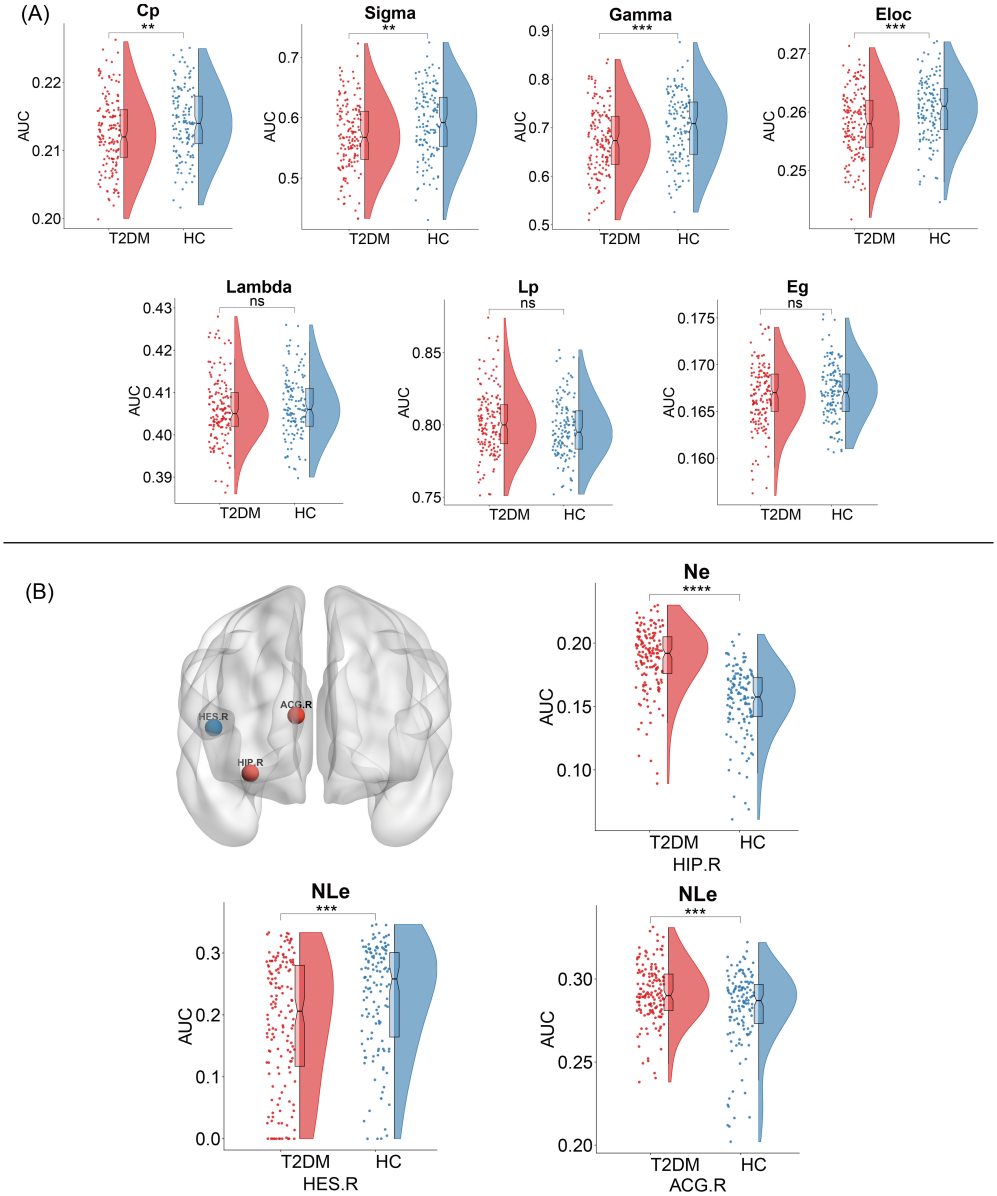
Topological changes in individual-based morphological brain network between the two groups. **(A)** Alterations in global profiles of the morphological brain network. Cp, clustering coefficient; Sigma (*σ*), small-world parameters; Gamma (*γ*), normalized clustering coefficient; Eloc, local efficiency; Lambda (*λ*), normalized characteristic path length; Lp, characteristic path length; Eg, global efficiency. **(B)** Alterations in nodal profiles of the morphological brain network. Red nodes represent nodal efficiency or nodal local efficiency increase, and blue nodes represent nodal local efficiency decrease, and the result is corrected using FDR. NE, nodal efficiency; NLe, nodal loca efficiency; HIP.R, right hippocampus; ACG.R, right anterior cingulate gyrus; HES.R, right transverse temporal gyrus; PHG.L, left parahippocampal gyrus.

Within the defined thresholds, both T2DM and HC exhibited small-world topologies (*γ* > 1, *λ* ≈ 1, *σ* = γ/λ > 1) in the Individual-based morphological brain network constructed based on the JSD method. Compared to HC, global attributes of T2DM were significantly reduced, including Cp (*p* = 0.03), γ (*p* = 0.0009), σ (*p* = 0.02), and Eloc (*p* = 0.0009), with statistically significant between-group differences (*p* < 0.05). Although λ (*p* = 0.4), Eg (*p* = 0.0812) decreased and Lp (*p* = 0.067) increased in the T2DM group compared to HCs, the difference between groups was not statistically significant. The above details are summarized in Figure 3A. The results based on the KLD network are consistent with those based on the JSD network, as detailed in Supplementary Figure 1A for details.

### 3.4 Alterations in nodal profiles of morphological brain network

We identified several regions that showed significant differences in node properties (*p* < 0.05, FDR corrected), with increased nodal efficiency in the right hippocampus, slightly increased nodal local efficiency in the right anterior cingulate gyrus, and reduced nodal local efficiency in the transverse temporal gyrus in patients with T2DM compared to HCs, and summarized the detailed information in Figure 3B. However, there was no significant difference in the number of betweenness centrality and degree centrality between the groups. Compared to the JSD-based network, the KLD-based network found that the brain region in which the nodal local efficiency was reduced changed from the right transverse temporal gyrus to the left parahippocampal gyrus, and the remaining nodes were the same, with reduced node efficiency in the right hippocampus, for further details, please refer to Supplementary Figure 1B.

### 3.5 Results of correlation analysis

**Figure 4:**
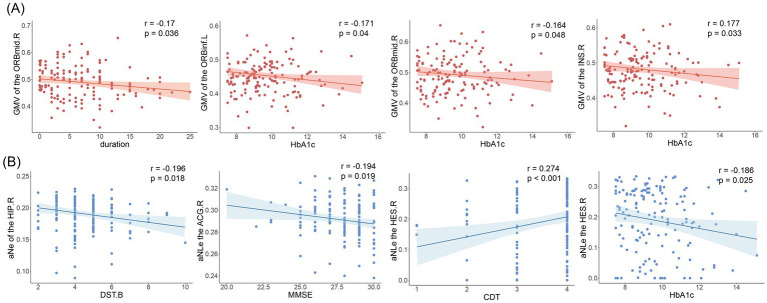
Results of correlation analysis. **(A)** Correlation of gray matter volume with disease duration and glycosylated hemoglobin. ORBmid.R, right orbitofrontal middle gyrus; ORBinf.L, left orbitofrontal inferior gyrus; INS.R, right insula. **(B)** Nodal efficiency and nodal local efficiency correlate with clinical indicators and cognitive scale scores. DST.B, Digit Span Test (backward); MMSE, mini-mental state examination; CDT, Clock-Drawing Test; HbA1c, glycosylated hemoglobin; Ne, nodal efficiency; NLe, nodal local efficiency.

Gray matter volume in the right orbitofrontal middle gyrus was negatively correlated with disease duration (*r* = −0.17, *p* = 0.036). There was a significant weak correlation between glycated hemoglobin and the left orbitofrontal inferior gyrus (*r* = −0.171, *p* = 0.04), right insula (*r* = −0.177, *p* = 0.033), and right orbitofrontal middle gyrus gray matter volumes (*r* = −0.164, *p* = 0.048), as shown in Figure 4A.

Correlation analysis showed that nodal efficiency of the right hippocampal in T2DM patients was negatively correlated with DST-B scores (*r* = −0.196, *p* = 0.018), nodal local efficiency of the anterior cingulate gyrus was negatively correlated with MMSE (*r* = −0.194, *p* = 0.019). Transverse temporal gyrus node local efficiency correlates with glycated hemoglobin (*r* = −0.186, *p* = 0.025) and CDT scores (*r* = −0.186, *p* = 0.025). As shown in Figure 4B. No significant correlation was found for the other indicators.

## 4 Discussion

Our study is the first to use both JSD and KLD methods to construct individual-level gray matter morphological brain networks in T2DM, along with VBM, and SBM analyses, and to obtain three main findings: compared to HCs, (1) reduced gray matter volume and cortical thickness in T2DM patients were mainly concentrated in the central control network. (2) In the individual morphologic brain networks, we found that the global properties of T2DM were significantly reduced, the integration and separation of networks were decreased, and the node properties were altered mainly in the right hippocampus, parahippocampal gyrus, temporal lobe, and cingulate gyrus. (3) Our brain morphology analyses (VBM and SBM) supported the results of the individual morphology network analyses, with overlapping brain regions, mainly in the limbic/paralimbic network, and correlations between some of the node attributes and cognitive and clinical indicators. These findings deepen our understanding of the large-scale neural network mechanisms of T2DM.

VBM and SBM are considered to be two complementary modalities of morphological analysis, making the results more reliable and comprehensive (14). Our findings based on these two methods show extensive cortical atrophy in patients with T2DM. Although the atrophied brain regions have different detailed names, they are mainly located in the frontal lobe, temporal lobe, dorsal parietal lobe, cingulate gyrus, and insula, which is largely consistent with previous studies (41–43). These brain regions are mainly located in the central executive network, which plays a central role in cognitive control and executive function (44). Some studies have shown disrupted functional connectivity of the central executive network in patients with T2DM (45), all of which suggest that the central executive network is impaired in patients with T2DM and may be related to the development of cognitive impairment. Meanwhile, our study also found a negative correlation between gray matter volume and glycosylated hemoglobin in some brain regions, suggesting that the higher the blood glucose, the more severe the cortical atrophy. Long-term hyperglycemia is an independent factor in cognitive decline in diabetic patients (46). Therefore, we hypothesized that cortical atrophy may be a mediating variable in the relationship between hyperglycemia and cognitive decline. Future subgroup analyses (with or without cognitive impairment), mediation analyses, and longitudinal analyses of T2DM are needed to clarify the relationship.

In JSD-based and KLD-based networks, T2DM showed lower Cp, *σ*, *γ*, *λ*, Egloc, Eg, and longer Lp compared with HCs, implying that normal small-world organization is disturbed, affecting brain functional separation and integration (47). Previous structural covariance network studies based on cortical thickness and white matter network studies have found similar topological alterations in type 2 diabetes (15, 19, 48). Differently, although our study showed a trend toward increased Lp (*p* = 0.065) and decreased Eg (*p* = 0.0812), the differences were not statistically significant. This could be due to our relatively young participant group (mean age 50.54 years) and their short disease history (median duration 5 years). Overall, the topological network of T2DM patients is generally impaired, leading to a decrease in the speed of information processing and in the network’s resistance to external disturbances, which may be related to cognitive and memory decline. Notably, our study also revealed altered local efficiency of nodes in the anterior cingulate gyrus and the transverse temporal gyrus, as well as reduced gray matter volume in the anterior cingulate gyrus and cortical thickness in the superior temporal gyrus [the transverse temporal gyrus and the superior temporal gyrus are anatomically adjacent and functionally synergistic (49)], so we found that the results of the network analysis partially overlapped with the results of the morphometric analysis. This suggests that the anterior cingulate gyrus and temporal lobe play an important role in T2DM. Furthermore, we speculate that the altered topological network properties might be related to gray matter atrophy (50, 51).

The cingulate, hippocampus, and parahippocampal gyrus all belong to the limbic/paralimbic system, which plays a key role in long-term memory, attention, and emotion regulation. Previous studies have shown that these sites are known to be associated with T2DM (52, 53), supporting our results. Our findings of increased nodal efficiency in the right hippocampus and nodal local efficiency in the anterior right cingulate gyrus in patients with T2DM may seem strange. However, there are also functional connectivity networks that show increased degree centrality of nodes in the hippocampus and anterior cingulate gyrus in patients with T2DM (18), and the classical explanation for this finding is a compensatory mechanism (54). Meanwhile, in our study, nodal efficiency in the right hippocampus was negatively correlated with DST-B scores reflecting working memory (31), and nodal localization efficiency in the anterior cingulate gyrus was negatively correlated with MMSE scores. We hypothesize that the brain undergoes compensatory changes in order to maintain normal cognition in the pre-disease phase, but that cognitive levels eventually decline as the disease progresses. Therefore, we speculate that compensatory changes in the properties of nodes in the topological network may be an indicator for early detection of cognitive changes and that the hippocampus and anterior cingulate gyrus may be imaging markers for identifying cognitive impairment in T2DM (55).

Nodal local efficiency reflects the efficiency of information communication between the neighbors of the node after removing the node, reflecting the separation of local information and the fault tolerance of the sub-network. Our study found that decreased nodal local efficiency of the transverse temporal gyrus was associated with elevated glycosylated hemoglobin and reduced CDT scores. One study has shown that the performance of CDT in detecting cognitive impairment is closely related to the temporoparietal cortex (56), consistent with our study. The transverse temporal gyrus is primarily responsible for auditory processing and semantic comprehension. Therefore, we suggest that in the presence of long-term chronic hyperglycemia, the structural network of the brain in T2DM patients is less fault-tolerant, which may lead to decreased auditory and semantic comprehension. The KLD-based network showed reduced nodal local efficiency in the parahippocampal gyrus, which is closely related to the hippocampus (57), and both parahippocampal and hippocampal subregions contribute uniquely to the encoding, consolidation, and retrieval of declarative memory. The brain insulin receptor signaling pathway (IRSP) is present in the parahippocampal gyrus (58), which helps to control processes such as synaptic plasticity, neuroprotection, survival, growth, and energy metabolism (59), which are all related to cognition. However, insulin resistance, amyloid *β* (Aβ) deposition, and hyperphosphorylation of Tau lead to disruption of cerebral insulin signaling (60), which may account for the higher risk of dementia and reduced local efficiency of the parahippocampal gyrus node in patients with T2DM.

There are some limitations to this study. First, the correlation results in our study were all weak but could suggest the existence of such a trend, possibly due to the relative youth of our patients and the relatively short duration of the disease. Second, previous studies have reported some effects of anti-glycemic drugs on the brain (61), and the effect of drugs on certain outcomes cannot be ruled out in our study. In addition, although we excluded some metabolic diseases from the exclusion criteria, we still cannot rule out the influence of residual or unmeasured confounders on the experimental results. Finally, because the present study was like a cross-sectional study and had a relatively small sample size that did not allow for causal inferences, future longitudinal studies examining alterations in neural networks in patients with type 2 diabetes are needed to assess the relationship between abnormal brain alterations and cognition during disease progression.

## 5 Conclusion

In conclusion, our morphological analyses suggest extensive cortical atrophy in patients with T2DM. Individual-based morphological brain network analysis revealed impaired network integration and separation. It also identified overlapping and cognitively relevant key brain regions, primarily within the limbic/paralimbic network (especially the hippocampus and cingulate gyrus), which may serve as imaging markers for identifying cognitive deficits in T2DM. These findings deepen our understanding of the mechanisms of large-scale neural networks in T2DM and provide additional theoretical support for exploring the relationship between T2DM and cognitive impairment.

## Data Availability

The raw data supporting the conclusions of this article will be made available by the authors, without undue reservation.

## Footnotes

^1^ https://www.nitrc.org/projects/cat/

^2^ https://www.fil.ion.ucl.ac.uk/spm/software/spm12/

^3^ http://surfer.nmr.mgh.harvard.edu/

^4^ https://www.nitrc.org/projects/gretna/

^5^ https://www.mathworks.com/products/new_products/release2022b.html
