# Supplementary material for: Association of individual-based morphological brain network alterations with cognitive impairment in type 2 diabetes mellitus
Source: Front Neurol. 2025 Jan 9;15:1519397. doi: 10.3389/fneur.2024.1519397 (PMC11754055; doi:10.3389/fneur.2024.1519397)
Supplement: Supplementary file 1 [file Data_Sheet_1.pdf]

Supplementary Material

1 Supplementary Figures

Supplementary Figure 1. Topological changes in morphological brain network based on KLDs method. (A) Alterations of global topological properties between T2DM and HCs. (B) Alterations of nodal topological properties between T2DM and HCs.
