# Supplementary figures and images for: Association of individual-based morphological brain network alterations with cognitive impairment in type 2 diabetes mellitus
Source: Front Neurol. 2025 Jan 9;15:1519397. doi: 10.3389/fneur.2024.1519397 (PMC11754055; doi:10.3389/fneur.2024.1519397)

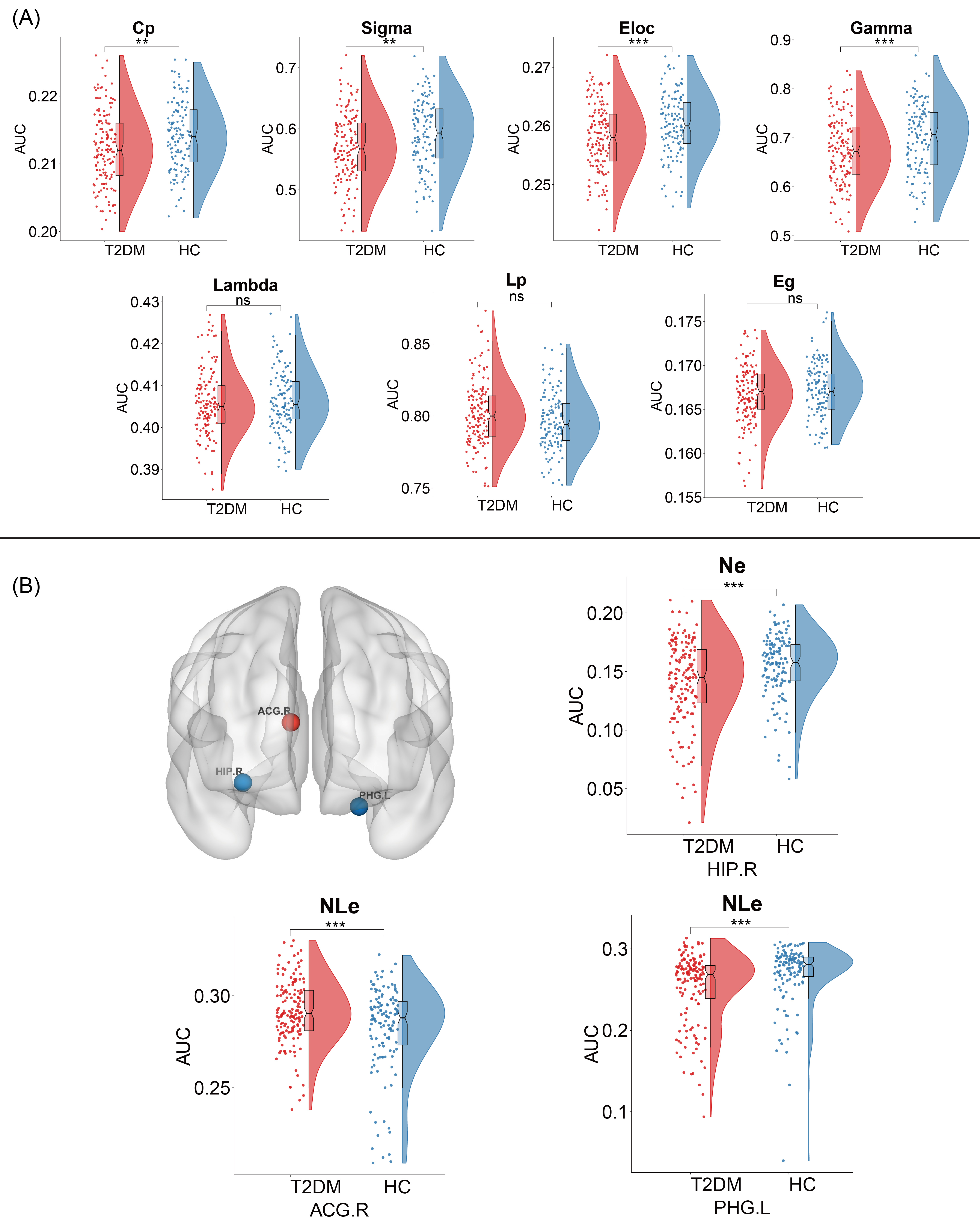

Supplement: Supplementary file 2 [file Image_1.jpeg]
